# Proper Treatment and Management of Postcardiotomy Refractory Vasospasm

**DOI:** 10.1155/2023/9988680

**Published:** 2023-11-10

**Authors:** Caleb R. Weissman, Stephane Leung Wai Sang

**Affiliations:** ^1^College of Human Medicine, Michigan State University, USA; ^2^Division of Cardiothoracic Surgery, Meijer Heart and Vascular Institute, Center for Structural and Transcatheter Heart Valve Therapies, Corewell Health, Grand Rapids, Michigan, USA

## Abstract

We present here a unique case in which a 63-year-old man developed diffuse coronary vasospasm on postoperative day (POD) 1 following uneventful aortic valve replacement, replacement of ascending aorta, and coronary artery bypass. Subsequent emergent coronary angiogram demonstrated diffuse native coronary artery vasospasm that was only transiently responsive to intracardiac nitroglycerin, resulting in persistent cardiogenic shock and severe biventricular dysfunction. The patient was, thus, placed on femoral-femoral venoarterial (VA) extracorporeal membrane oxygenation (ECMO) with Impella support. This strategy allowed the weaning of vasopressors and enabled the resolution of the vasospasm. The patient was ultimately discharged on POD 17 without further complications. This case demonstrates our management strategy to provide life-saving support for patients facing postcardiac surgery refractory vasospasm.

## 1. Introduction

Refractory coronary vasospasm after coronary artery bypass graft (CABG) is a rare and life-threatening complication that is estimated to occur in 0.43% to 1.3% of cases [1, 2]. In the largest published case series of such patients, the mortality rate for diffuse coronary vasospasm was 71% [1]. Additionally, there is a distinct lack of consensus on the management of these patients after surgery, though prompt diagnosis and supportive care with the use of peripheral vasodilators and mechanical support seem to be paramount [3–5]. While the precise mechanism of refractory vasospasm is unknown, the phenomenon is likely induced by the coronary response to both physiologic stress and spasmogen stimulation [2, 6]. The physiologic stress of surgery results in both mechanical and nervous stimulation of the coronaries [7]. Spasmogens are vasoconstrictors that include chemicals endogenously released when under stress from the endothelium (endothelin-1, thromboxane A_2_, etc.), platelets (5-hydroxytryptamine), and mast cells (histamine) [2, 7]. We present here a unique case in which the patient developed diffuse coronary vasospasm following heart valve surgery, almost 24 hr later.

## 2. Case Presentation

A 63-year-old male with history of bicuspid aortic valve, ascending aortic aneurysm, chronic obstructive pulmonary disease, diabetes mellitus, hypertension, obesity, and previous tobacco abuse developed moderate aortic valve stenosis. Preoperative echocardiography revealed an aortic valve mean gradient of 22 mm Hg with mild aortic insufficiency, velocity of 3.1 m/sec, and an aortic valve area of 1.1 cm^2^. Computed tomography imaging demonstrated a 5.2 cm ascending aorta which had grown 5 mm over the previous 12 months. Preoperative coronary angiogram showed a 90% right coronary lesion.

The patient underwent uneventful aortic valve replacement with a 23 mm Edwards Inspiris valve, replacement of the ascending aorta with a 30 mm Gelweave graft, and a single bypass with a saphenous vein graft to the posterior descending artery. The patient was weaned off cardiopulmonary bypass with low-dose norepinephrine and epinephrine. The patient was extubated on postoperative day 0 but required low-dose norepinephrine for persistent vasoplegia.

**Figure 1:**
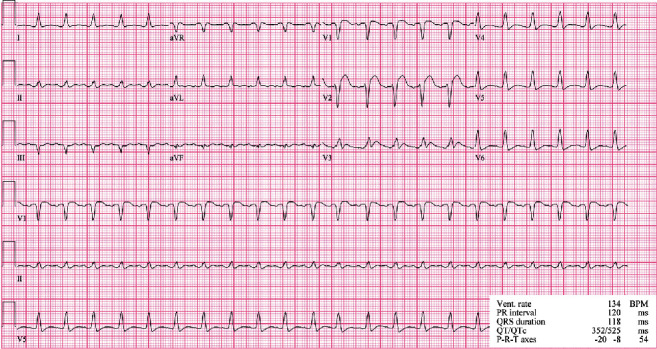
12-lead ECG that was taken during the vasospastic event. Findings indicated ST elevation in anterior leads V1-V3.

**Figure 2:**
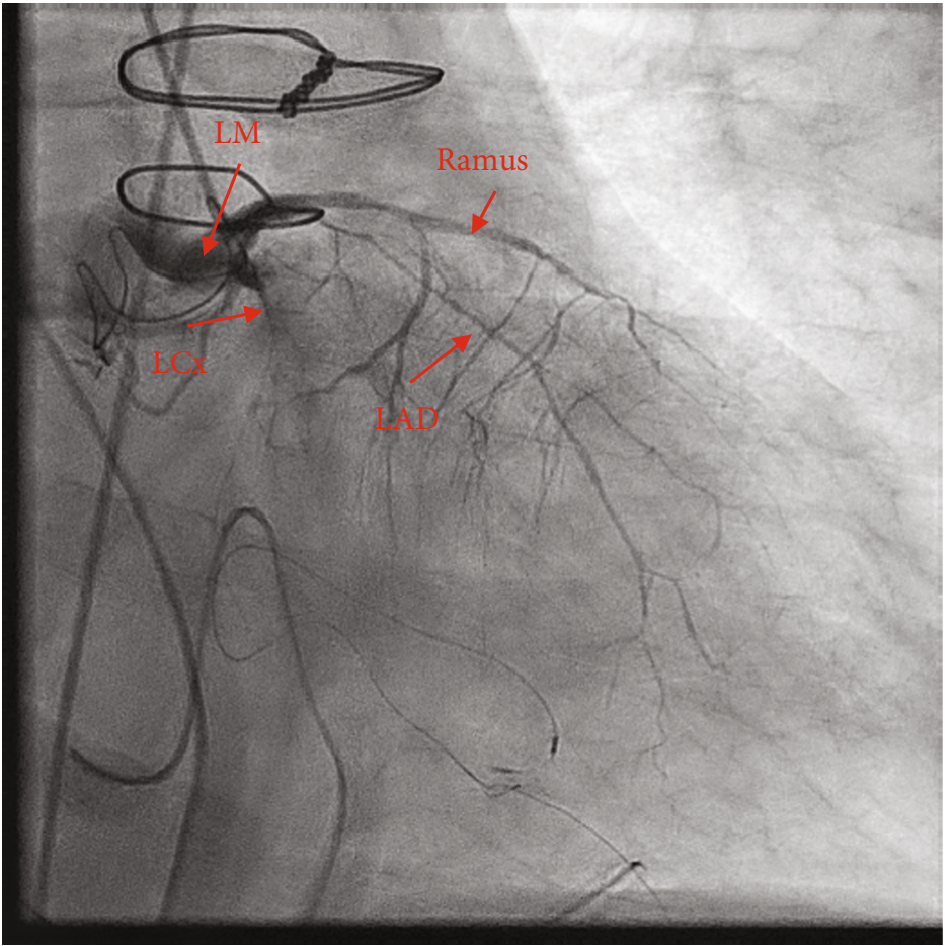
Coronary angiogram of vasospasm of the left main coronary artery and its tributaries. LM: left main; LCx: left circumflex; LAD: left anterior descending; Ramus: ramus intermedius.

**Figure 3:**
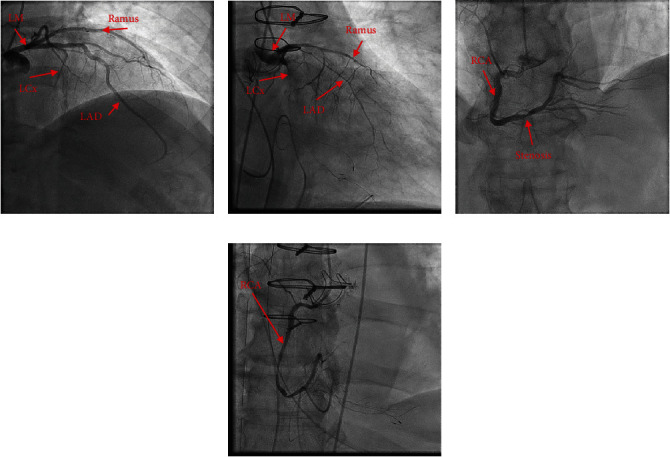
Angiogram of coronary vessels before and after vasospasm. Coronary angiogram of the left main coronary artery (a) and right coronary artery (c) preoperatively. Coronary angiogram of the left main coronary artery (b) and the right coronary artery (d) postoperative day 1. LM: left main; LCx: left circumflex; LAD: left anterior descending; Ramus: ramus intermedius; RCA: right coronary artery.

**Figure 4:**
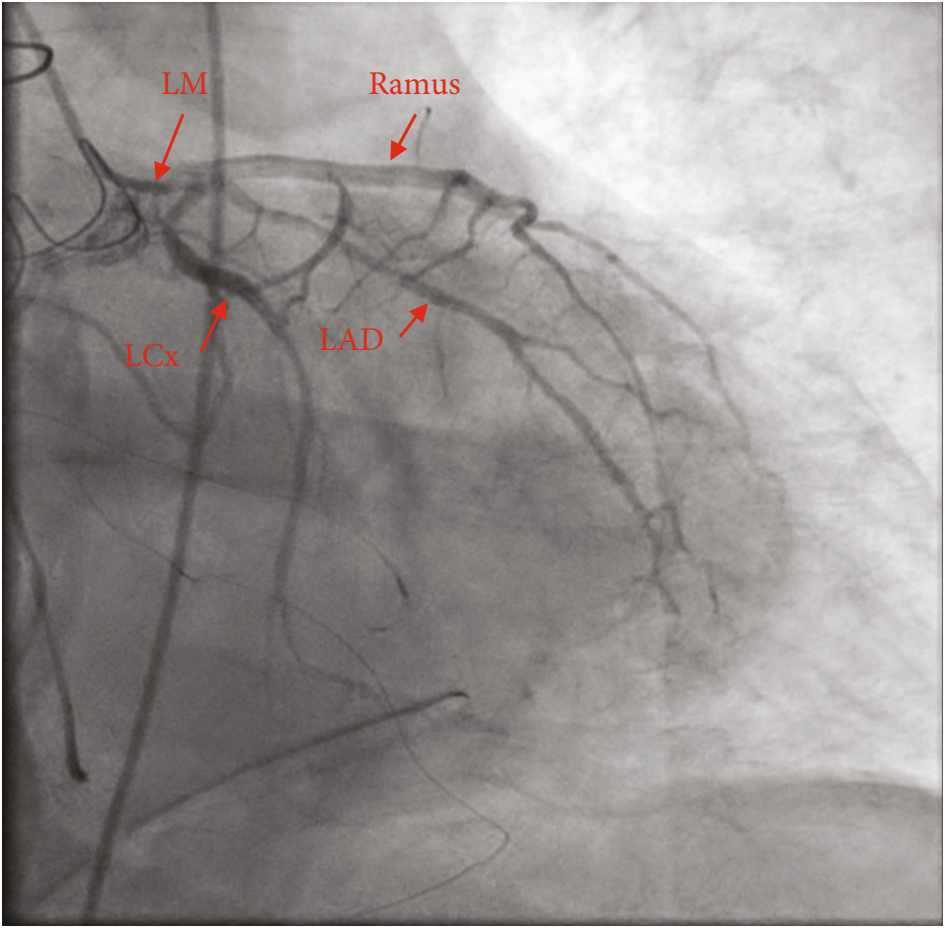
Coronary angiogram of the left main coronary artery and its tributaries after intracoronary administration of nitroglycerin. LM: left main; LCx: left circumflex; LAD: left anterior descending; Ramus: ramus intermedius.

On the morning of postoperative day 1, he went into cardiac arrest, requiring CPR and reintubation. Electrocardiogram demonstrated ST elevations in leads V1 and V2 (Figure 1), while transesophageal echocardiogram (TEE) revealed right ventricular and left ventricular inferolateral and apical wall hypokinesis (Supplement 1) with an estimated left ventricular ejection fraction (LVEF) of 30%. The patient had subsequent return of spontaneous circulation but continued to have multiple rhythm changes with intermittent loss of pulse. Given his continued hemodynamic instability and concerns for cardiac ischemia, the decision was made to initiate femoral-femoral VA-ECMO to facilitate left heart catheterization. Immediate repeat coronary angiogram showed a patent saphenous vein graft to the right coronary artery; however, all the native coronary vessels were severely and diffusely narrowed by spasms (Figures 2 and 3). Nitroglycerin injected directly into the left main coronary artery transiently resolved the vasospasm (Figure 4) with improvement in left ventricular function; however, these effects were transient; therefore, VA-ECMO was continued, and an Impella 3.5 was placed across the aortic valve to help wean vasopressor requirements.

On postoperative day 3, with full mechanical support and intravenous calcium channel blockers, repeat imaging was consistent with biventricular recovery (Supplement 2), so the patient was decannulated from ECMO. The patient was ultimately discharged home on postoperative day 17 in stable condition without further complications. On postoperative day 24, outpatient TTE revealed ventricular recovery with an LVEF of 55% with no aortic insufficiency, an aortic valve mean gradient of 13 mm Hg, a maximum gradient of 27.5 mm Hg, velocity of 2.62 m/sec, and an aortic valve area of 1.6 cm^2^. The ascending aorta above the sinotubular junction had a diameter of 3.2 cm.

## 3. Discussion

In this case, the primary cause of vasoconstriction was elevated endogenous and exogenous vasoconstrictors; however, this phenomenon was likely multifactorial and also due to a mix of mechanical vascular injury and oxidative stress [3]. Postoperatively, our patient remained on a norepinephrine drip, and this, in combination with the patient's initial vasoplegic state, may have contributed to the delayed onset of vasospasm. On cardiac arrest, the patient was still on vasopressors and the stress from cardiac ischemia, and subsequent resuscitation likely contributed to the onset of vasospasm.

Once the vasoconstriction did occur, our strategy was to maintain the patient's hemodynamic stability and coronary blood flow. We aimed to reduce the ischemic stress experienced by the myocardial tissue via comprehensive cardiovascular support. Sambuceti et al. found that in patients with coronary artery disease, there can be an inappropriate vasoconstrictive response to myocardial ischemia due to increased metabolic demand [8]. This phenomenon may have contributed to the transient nature of the patient's vasospasm.

This case of coronary vasospasm occurring almost 24 hours after cardiac surgery is the first of its kind to be described. From a review of the literature, vasospasm associated with CABG has been observed to occur intraoperatively [4] or in the immediate hours after the operation [1–3, 6]. Our case reveals that prompt ECMO initiation and full biventricular support with early diagnosis by repeat heart catheterization may help to prevent the high mortality rate of this complication.

## Supplementary Materials

Supplementary Materials Supplementary Figure 1: transesophageal echocardiogram (TEE) revealing right ventricular and left ventricular inferolateral and apical wall hypokinesis with an estimated left ventricular ejection fraction (LVEF) of 30%. Supplementary Figure 2: after the resolution of vasospasm, transesophageal echocardiogram (TEE) reveals normal right ventricular systolic function. The left ventricle is hyperdynamic with an estimated ejection fraction of 65% and no regional wall motion abnormalities.
